# Artificial intelligence cooperation and domestic absorptive capacity: evidence from ASEAN economies

**DOI:** 10.1098/rsos.261565

**Published:** 2026-10-07

**Authors:** Quanjun Zhang, Jian Chen, Yang Zhao

**Affiliations:** ^1^ China-ASEAN School of Economics, Guangxi University, Nanning, People's Republic of China; ^2^ Department of Geography, University College London, London, UK; ^3^ School of Law, Southwest University of Science and Technology, Mianyang, Sichuan, People's Republic of China

**Keywords:** bilateral AI cooperation, absorptive capacity, ASEAN, technology diffusion

## Abstract

Cross-border artificial intelligence (AI) cooperation is expanding, but evidence on its effect on domestic technological capability in recipient economies is scarce. Drawing on absorptive capacity theory, we create a composite AI absorptive capacity index for 10 Association of Southeast Asian Nations (ASEAN) economies, encompassing innovation foundations, application foundations and industrial support. We measure bilateral AI cooperation intensity via co-publications, co-patents and investment linkages. Using a 2015–2025 bilateral panel with fixed-effects and three-way fixed-effects models, we find a positive association between cooperation intensity and absorptive capacity, robust across all three index dimensions and to sample restrictions and winsorization. Heterogeneity analysis shows that China-related cooperation offers no general premium. A more favourable link emerges only in economies with weaker initial industrial foundations, and it is fragile under stricter sample restrictions. The findings imply that the developmental value of AI cooperation hinges not on cooperation volume but on embedding external knowledge into domestic learning, application and industrial adaptation systems.

## 1. Introduction

Artificial intelligence (AI) has moved from a frontier technology to a central element of development strategy across the Asia-Pacific. Governments in the region increasingly view AI as an input into industrial upgrading and digital transformation, and cross-border cooperation has become a primary channel through which AI-related knowledge, technical resources and application experience circulate across economies [1,2]. China–ASEAN cooperation in digital infrastructure, smart-city initiatives and AI-related research has expanded considerably in recent years [3,4]. Yet the deepening of cooperation does not automatically imply the building of domestic capability. The gains from external AI inputs depend critically on the receiving economy's knowledge stock, human capital, digital infrastructure and industrial support systems. Association of Southeast Asian Nations (ASEAN) economies differ greatly along each of these dimensions: Singapore and Malaysia possess well-established innovation systems, Vietnam and Indonesia combine large domestic markets with expanding application scenarios, while Cambodia, Myanmar and Laos face tighter constraints in research capacity and institutional readiness. The same external cooperation input can yield very different domestic outcomes. This tension provides the motivating problem of the paper.

Two strands of prior work are relevant but leave an empirical gap. The first examines AI adoption at the firm level. Babina *et al.* [5] show that AI adoption is associated with product innovation and firm growth, particularly where complementary data and human capital endowments exist. Han *et al.* [6] find that AI application pilot zones in China improve urban innovation performance. Liu *et al.* [7] link AI capability to multi-dimensional intellectual capital and organizational agility. These studies establish that AI generates economic returns, but they operate at the firm level. The second strand examines absorptive capacity in the context of foreign direct investment (FDI) and technology diffusion. Keller [8] provides the benchmark framework linking trade and FDI to international technology diffusion, with domestic absorptive conditions determining realized productivity gains. Ferraz *et al.* [9], Zhang & Liu [10] and Yang *et al.* [11] show that FDI spillovers on regional innovation and economic complexity depend on local absorptive capacity. Kastelli *et al.* [2] document that digital capacity affects innovation through the mediating role of absorptive capacity. These studies establish that external knowledge is not a free good, but they focus on market-mediated channels, primarily FDI and trade. Whether policy-mediated bilateral cooperation, which bundles knowledge exchange, collaborative production and institutional partnership in ways that FDI alone does not, is associated with domestic capability formation remains an unanswered empirical question.

This study examines whether stronger bilateral AI cooperation is associated with stronger absorptive capacity in ASEAN economies, and whether this association differs for China-related cooperation and varies with the receiving economy's initial industrial conditions. Drawing on absorptive-capacity theory, we construct a composite index of AI absorptive capacity covering three dimensions: innovation foundations, application foundations and industrial support conditions. Bilateral AI cooperation intensity is measured through co-publications, co-patents and AI-related investment linkages between ASEAN economies and their external partners, primarily using data from the Emerging Technology Observatory at Georgetown University. A bilateral panel spanning 2015 to 2025 with fixed-effects specifications is employed to estimate whether cooperation intensity is associated with domestic capability formation and whether this association is heterogeneous across partners and initial conditions.

The paper makes two contributions. First, it extends the study of AI from firm-level adoption to the question of how bilateral cooperation relates to country-level capability formation. Second, by linking bilateral cooperation rather than FDI or trade to absorptive capacity, it opens empirical inquiry at the intersection of the absorptive-capacity literature, the regional cooperation literature and the emerging work on AI and development. The focus on ASEAN, with its considerable heterogeneity in digital readiness and industrial conditions, provides a setting in which the conditional logic of absorptive capacity can be tested against observable variation.

The remainder of the paper is organized as follows. Section 2 reviews the absorptive-capacity literature and develops the hypotheses. Section 3 describes the data, variable construction and empirical strategy. Section 4 presents the baseline estimates, heterogeneity analysis, dimension-level results and robustness checks. Section 5 concludes with policy implications and limitations.

## 2. Literature review and hypotheses

### 2.1. Absorptive capacity and cross-border AI cooperation

Absorptive-capacity theory provides a foundational lens for understanding whether external technological linkages translate into domestic capability building. Cohen & Levinthal [1] define absorptive capacity as the ability to recognize the value of external knowledge, assimilate it and apply it to commercial ends. Zahra & George [12] extend this concept into a dynamic capability process encompassing acquisition, assimilation, transformation and exploitation. Subsequent work has generalized absorptive capacity from the firm level to broader regional and national contexts, where the construct includes not only organizational routines, but also the wider conditions that enable the reception, processing and productive use of external knowledge, such as human capital, digital infrastructure, institutional support and industrial ecosystems [13]. A substantial body of prior literature has examined knowledge spillovers from FDI, establishing that the benefits of external technological linkages are contingent upon domestic absorptive capacity. Gereffi *et al.* [14] and Pietrobelli & Rabellotti [15] demonstrate that the potential for knowledge spillovers is highly unevenly distributed across economies, depending on differences in learning capacity, industrial structure and institutional readiness. This literature provides an important foundation for understanding technology diffusion. However, it predominantly addresses conventional technology transfer, which involves codified technologies that can be transferred through equipment, manuals or turnkey projects. While we do not test the specific mechanism through which cooperation differs from FDI, the conceptual case for treating cooperation as a distinct analytical channel rests on several features of AI as a general-purpose technology. Recent work characterizes AI as a general-purpose technology whose economic manifestations depend critically on complementary intangible investments, organizational adjustment and the extent to which firms and economies systematize new tools as part of their production and decision-making processes [5,16]. Unlike conventional technologies, AI is data-driven and ecosystem-embedded, and its effective use presupposes access to computing infrastructure, availability of skilled personnel, institutional support for data governance and organizational capacity to integrate AI tools into production processes. Hassan *et al.* [17] and Kastelli *et al.* [2] show that external technological opportunities generate faster and more durable returns when regions possess local capacity to absorb and exploit them. Murad *et al.* [18] further demonstrate that digitalization and innovation linkages are conditional on the receiving economy's absorptive conditions. These studies suggest that the absorptive-capacity framework remains relevant for AI cooperation, but the mechanisms through which absorption occurs and the conditions required for successful absorption may differ from those identified in the traditional FDI-spillover literature. This paper builds on these insights by examining whether bilateral AI cooperation, measured through co-publications, co-patents and investment linkages, is associated with stronger domestic absorptive capacity in ASEAN economies. We argue that AI cooperation operates through multiple channels, including knowledge transmission via joint research, collaborative resources through co-patenting and capital-embedded technology transfer through investment linkages. Each channel provides opportunities for domestic actors to access, assimilate and build upon externally generated AI-related knowledge. However, the effectiveness of these channels is contingent upon domestic absorptive conditions. On this basis, we propose our first hypothesis:

H1. Stronger bilateral AI cooperation is associated with higher AI absorptive capacity in ASEAN economies.

### 2.2. Initial industrial conditions and heterogeneous capability effects

The relationship between bilateral AI cooperation and domestic absorptive capacity is unlikely to be uniform across ASEAN economies. A central proposition in absorptive-capacity theory is that the gains from external knowledge linkages are conditional on prior domestic capabilities [1]. Economies with stronger initial industrial foundations, including broader manufacturing bases, denser innovation networks and more established digital infrastructure, possess greater capacity to identify, assimilate and exploit externally generated knowledge. Conversely, economies with weaker initial industrial foundations face tighter constraints in research capacity, industrial support systems and institutional readiness, which may limit their ability to absorb and build upon external AI cooperation. Two competing theoretical expectations emerge regarding how initial industrial conditions moderate the cooperation–absorptive capacity relationship. The complementary-assets argument posits that the benefits of external AI cooperation are larger in economies with stronger initial industrial foundations. These economies possess the complementary assets necessary to absorb and exploit external AI-related knowledge, including skilled personnel, digital infrastructure and supportive institutions [5,17]. In this view, AI cooperation reinforces existing capabilities and enables high-end coordination and selective upgrading within systems that are already reasonably deep. The compensatory role argument, by contrast, posits that external AI cooperation may have larger marginal effects in economies with weaker initial industrial foundations. In these economies, domestic absorptive capacity is constrained by missing or underdeveloped foundational conditions. External AI cooperation can compensate for these deficiencies by providing access to critical technical interfaces, application pathways and supporting resources that are difficult to access through domestic channels alone [9,19,20]. AI cooperation, in this view, performs a compensatory function: it eases capability shortages and opens access to knowledge and application resources that would otherwise remain out of reach. The net effect is ultimately an empirical question. However, the compensatory role argument may be particularly relevant in the ASEAN context for two reasons. First, the region exhibits sharp disparities in initial industrial foundations. Singapore and Malaysia entered the current digital transition with relatively well-established digital infrastructure and innovation systems [21]. Vietnam and Indonesia possess large market sizes and expanding application scenarios but face constraints in high-end research capacity and industrial support systems. Other ASEAN members, such as Cambodia, Laos and Myanmar, confront more fundamental constraints in human capital, institutional readiness and digital infrastructure [22,23]. The same external AI cooperation input can therefore yield wildly disparate domestic outcomes across these economies. Second, AI is a rapidly evolving general-purpose technology. Latecomer economies may be able to leapfrog certain stages of industrial development by accessing AI-related knowledge and resources through bilateral cooperation, without having to first build every component of the innovation ecosystem domestically [24]. This logic is particularly relevant for China–ASEAN linkages. China has become an increasingly salient regional actor in digital cooperation, engaging in digital infrastructure projects, technology cooperation initiatives and smart-city development across ASEAN [4]. For ASEAN economies with weaker initial industrial foundations, China-related AI cooperation may provide access to technical interfaces, application experience and industrial support that are otherwise difficult to obtain. For economies with stronger initial industrial foundations, China-related cooperation may matter more for high-end coordination and selective upgrading. On this basis, and recognizing that the net effect is ultimately empirical, we propose our second hypothesis:

H2. The positive association between China-related bilateral AI cooperation and AI absorptive capacity is stronger in ASEAN economies with weaker initial industrial foundations.

## 3. Data, variables and empirical strategy

### 3.1. Data and sample

The empirical analysis uses a bilateral panel covering 10 ASEAN economies over the period 2015–2025. The unit of observation is an ASEAN economy–partner country–year triple. This structure allows us to link country-level absorptive-capacity outcomes to partner-specific exposure to AI cooperation. Formally, the same ASEAN country-year absorptive-capacity outcome is matched with different bilateral cooperation intensities across partner country observations. We cluster standard errors to account for the dependence induced by this structure at the country-year level.

The bilateral AI cooperation data are taken largely from the Emerging Technology Observatory (ETO) at Georgetown University and include cross-border co-publications, co-patents and investment linkages in AI-related activities. Macroeconomic and institutional control variables are taken using a variety of standard sources. Data on trade openness, for example, are taken largely from the World Bank, while government effectiveness and logistics performance are taken from there as well. Internet-use indicators come from the International Telecommunication Union, while gross domestic product (GDP)-related data was compiled from official statistical sources for ASEAN economies. After harmonizing the variables and bringing them into a common layout, the final estimation sample contains 1397 observations.

Some control variables for 2023–2025 contain missing values owing to publication lags. To retain temporal continuity, we fill a small number of missing values through linear interpolation for continuous series and trend-based extrapolation for variables with a clear pre-2023 trajectory. This treatment applies only to a subset of control variables and does not touch the dependent variable or the core explanatory variable. We report robustness checks that keep the sample to the observed-data period so that the baseline association can be compared to a more conservative sample window. A total of four control variables had missing values in 2024–2025, for example, trade openness (four missing country-years, 0.3% of the estimation sample), government effectiveness (six missing, 0.4%), foreign direct investment (six missing, 0.4%) and GDP per capita (three missing, 0.2%). Missing observations were filled using the last-observation-carried-forward method for variables with stable within-country trends (trade openness, government effectiveness) and linear interpolation for GDP-related variables where the pre-2023 trajectory was clearly monotonic. The total share of interpolated values in the estimation sample is below 0.5%. To assess whether imputation drives the results, we report a restricted-sample specification (2015–2023 in §4.5) that excludes all imputed observations.

### 3.2. Variable construction

#### 3.2.1. Dependent variable

The dependent variable is AI absorptive capacity, structured to measure the degree to which ASEAN economies can take the knowledge, technology and resourcing made available via cross-border AI cooperation into domestic innovation, application and industrial-support capability. The variable is constructed in line with the absorptive-capacity literature, including studies nowadays measuring capability conditions using multi-dimensional composite indicators [7,25]. The use of a composite index is part of the wider methodological rationale in multi-dimensional measurement for using the relationship between related indicators to arrive at a single standardized measure of the latent construct of the variable being assessed [26,27]. The index is made up of three dimensions.

The first dimension is innovation foundations. It captures the domestic factors that support knowledge input, human-capital accumulation and innovation-resource allocation. The indicators used in this dimension are research and development (R&D) expenditure as a share of GDP, education expenditure as a share of GDP and gross tertiary enrolment.

The second dimension is application foundations. It captures the digital-access and technological-readiness factors that support the use of the technology in real-life applications. The indicators used here are Internet-use rates, fixed broadband subscriptions per 100 people and information and communication technology (ICT) frontier-technology readiness.

The third dimension is industrial support conditions. It is this dimension which is meant to reflect the broader industrial and knowledge-support ecosystem in which AI-related activity can be embedded. This is measured by ICT service exports, the overall logistics performance index and ICT-related publication output.

Since the underlying indicators are measured in distinctly different units, we first standardize all of the component variables. We then average the standardized indicators with equal weights within each dimension. The final absorptive-capacity index is simply the average of the three dimension-specific indices. Higher values indicate stronger domestic capability conditions for absorbing and availing of the AI-related knowledge and cooperation inputs sourced externally.

#### 3.2.2. Core explanatory variable

The core explanatory variable is bilateral AI cooperation intensity, which aims to capture the extent of AI-related cooperation between an ASEAN economy and external partner countries. Existing studies on cross-border technology linkages suggest that external cooperation typically occurs through several channels: joint production of knowledge, collaborative invention and capital-related connections [8,25]. Following this logic, the paper uses three bilateral AI cooperation indicators: co-publications, co-patents and AI-related investment linkages. As all three variables are right-skewed and contain some zeros, we apply the inverse hyperbolic sine transformation before standardizing various metrics. This is common practice in empirical work when variables combine skewness with zero-valued observations, since the inverse hyperbolic sine transformation behaves similarly to a logarithmic transformation over positive values while retaining zeros in the sample [28]. The three are then standardized and averaged for a composite indicator of bilateral AI cooperation intensity.

#### 3.2.3. Heterogeneity variables

To consider whether China-related cooperation differs from the average case, we introduce a dummy variable taking the value one when the partner country is China, else zero. A second dummy identifies ASEAN economies with weaker prior industrial foundations. This was coded using the initial-year value of the industrial-support dimension of the absorptive-capacity index—economies below the median for the whole sample are treated as low-initial-industrial-foundation cases. The use of this initial-year value is intended to capture starting conditions, while minimizing the likelihood that current capability outcomes are mechanically mixed with current exposure to AI cooperation. Using these two indicators, we generate the interaction terms needed to assess whether the relationship between bilateral AI cooperation and absorptive capacity differs according to country type, or whether any China-related differential association is more apparent in economies with weaker initial industrial support conditions.

#### 3.2.4. Control variables

The baseline regressions include four country-level controls: GDP per capita, trade openness, government effectiveness and foreign direct investment. GDP per capita is included in logarithmic form and captures broad differences in economic development. Trade openness and foreign direct investment reflect the degree of external economic integration and exposure to international capital. Government effectiveness is included to capture institutional quality and implementation capacity. These variables are standard controls in studies of technology diffusion, absorptive capacity and external-spillover effects, since the domestic returns to external knowledge may depend on development level, openness, institutional quality and the broader investment environment. [20,29]. Table 1 summarizes the definitions, measurements and data sources for all variables used in the analysis. To assess whether the three dimensions of absorptive capacity are sufficiently distinct to justify a composite index, we report the pairwise correlation matrix in table 2. All correlations are positive and statistically significant at conventional levels. This indicates that the dimensions are conceptually related, yet not redundant, supporting the use of an unweighted average to construct the composite index.

**Table 1.** Variable definition.

abbreviation	full name
AIAC	AI absorptive capacity
BAIC	bilateral AI cooperation intensity
INNOV	innovation foundations
APPL	application foundations
INDUS	industrial support conditions
CHN	china partner dummy
LOWIND	low initial industrial foundation dummy
LNGDPPC	log GDP per capita
OPEN	trade openness
GOVEFF	government effectiveness
FDI	foreign direct investment

**Table 2.** Correlation matrix of absorptive capacity dimensions.

	innovation foundation	application foundation	industrial support condition
**innovation foundation**	1.000	0.835^***^	0.580^***^
**application foundation**	0.835^***^	1.000	0.487^***^
**industrial support condition**	0.580^***^	0.487^***^	1.000

Notes: ***p < 0.01.

### 3.3. Empirical strategy

To examine the association between bilateral AI cooperation and domestic absorptive capacity, the paper estimates a fixed-effects specification of the following form:

$$\begin{array}{r} AIAC_{ipt}=\beta_{1}\;BAIC_{ipt}+X_{it}^{\prime }\gamma +\mu_{i}+\lambda_{p}+\tau_{t}+\varepsilon_{ipt}, \end{array}$$

where ***AIAC***_***ipt***_ denotes the absorptive-capacity index for ASEAN economy *i* in year *t*, matched to partner country *p*; ***BAIC***_***ipt***_ is bilateral AI cooperation intensity; **X**_*it*_ is the vector of country-level controls; ***µ***_***i***_ denotes ASEAN-country fixed effects; ***λ***_***p***_ denotes partner-country fixed effects and ***τ***_***t***_ denotes year fixed effects.

This specification focuses on whether stronger bilateral AI cooperation exposure is associated with stronger domestic absorptive capacity after accounting for time-invariant differences across ASEAN economies and partner countries, as well as common time shocks. Since the dependent variable is measured at the ASEAN country-year level and appears repeatedly across partner-country observations within the same country-year, the error structure is likely to display within-country-year dependence. For this reason, standard errors are clustered at the country-year level.

To examine heterogeneous associations linked to China and to weaker initial industrial conditions, the paper estimates an extended specification with interaction terms,

$$\begin{array}{rcl} {AIAC}_{ipt}= & & \beta_{1}\;BAIC_{ipt}+\beta_{2}\left(CHN_{p}\times BAIC_{ipt}\right)+\beta_{3}LOWIND_{i}+\beta_{4}\left(LOWIND_{i}\times BAIC_{ipt}\right)\\ & & +\beta_{5}\left(CHN_{p}\times LOWIND_{i}\times BAIC_{ipt}\right)+X_{it}^{\prime }\gamma +\mu_{i}+\lambda_{p}+\tau_{t}+\varepsilon_{ipt}, \end{array}$$

where ***CHN***_***p***_ is the China-partner dummy, and ***LOWIND***_***i***_ identifies ASEAN economies with weaker initial industrial foundations. The coefficient on the triple interaction captures whether the association between China-related bilateral cooperation and absorptive capacity is more positive in economies that began with weaker industrial support conditions.

This research uses a fixed-effects strategy that aims to identify conditional patterns of association. Several potential sources of endogeneity remain, including time-varying omitted variables, measurement error in the composite indices and the possibility that improvements in domestic capability make countries more attractive partners for cross-border AI cooperation. Accordingly, results here are best viewed as demonstrating patterned associations with fixed-effects controls in place. To further probe the endogeneity concern, we estimate a specification using the one-year lag of bilateral AI cooperation intensity. Lagging the cooperation term by one period addresses the contemporaneous reverse-causality channel while retaining the temporal precedence interpretation. Component-level regression analyses and robustness checks using sample restrictions and outlier handling further test for a stable pattern of results across different methods and approaches.

Prior to discussing the regression results, we should first highlight that ASEAN economies are not strictly tied to a singular bilateral relationship; rather, they are also part of a much larger network of external AI-related networks. These linkages are present from various partner countries and, therefore, do not distribute evenly throughout ASEAN economies. For example, Singapore and Malaysia are both central players in this externalized network of knowledge, while the other economies are receiving weaker and more fragmented linkages. Figure 1 portrays the generalized trend of knowledge flow from partners to major ASEAN members, which should serve as context for the regression analysis, which seeks to determine whether stronger bilateral cooperation leads to a stronger domestic absorptive capacity.

**Figure 1.**
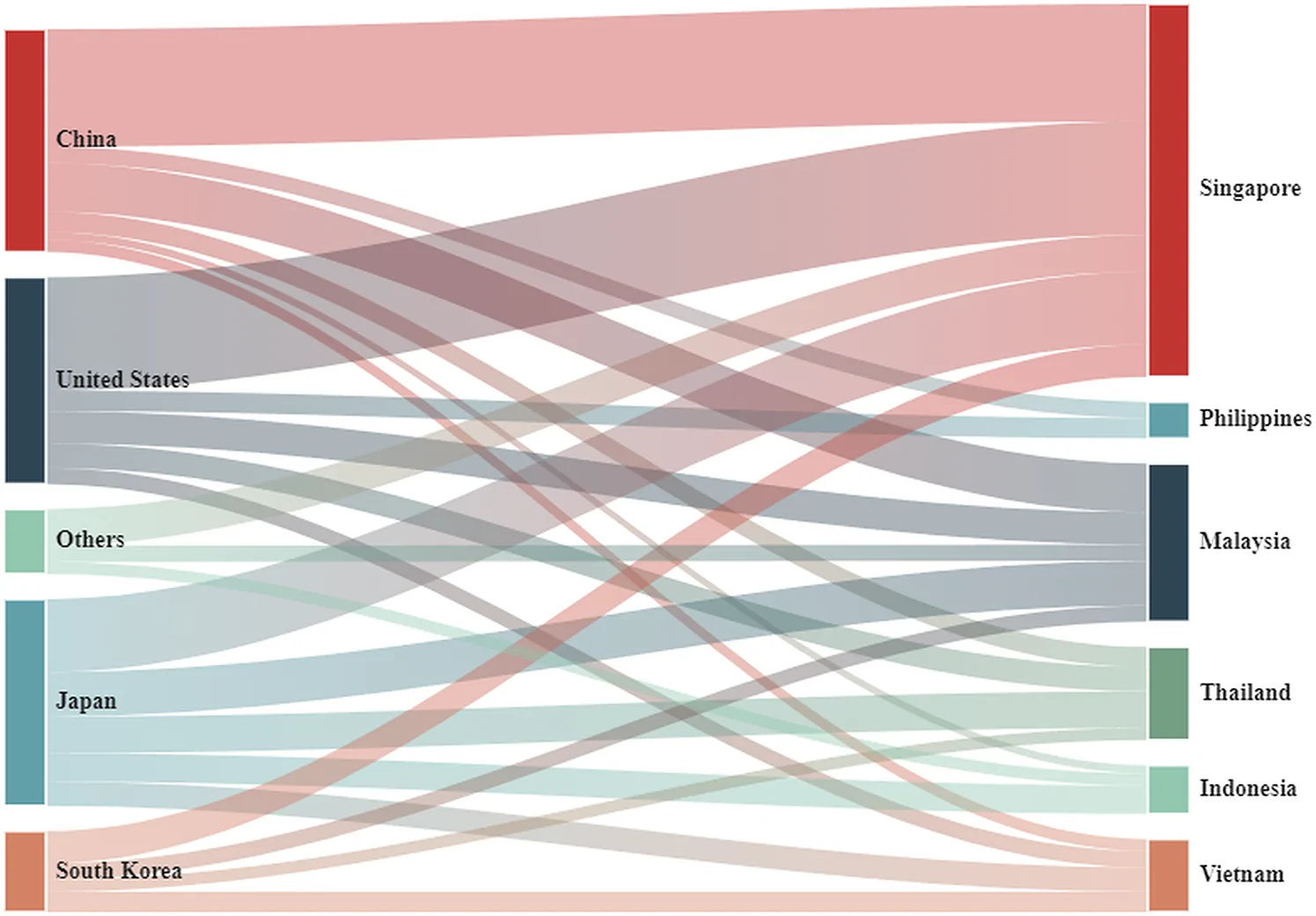
AI-related knowledge flows from partner countries to ASEAN economies.

## 4. Results

### 4.1. Descriptive patterns

Before turning to regression estimates, it is useful to examine the cross-country distribution of AI cooperation intensity and absorptive capacity across ASEAN economies. Figure 2 plots the position of each ASEAN member along these two dimensions for its cooperation with China. Singapore and Malaysia cluster in the upper-right region of the plot, with relatively high cooperation intensity and high absorptive capacity. Cambodia, Myanmar and Laos occupy the lower-left quadrant, with substantially lower scores on both measures. The middle-income ASEAN economies (Philippines, Vietnam, Indonesia and Thailand) fall between these two poles, showing a mixed pattern of cooperation exposure and domestic capability. The raw scatter plot is broadly consistent with a positive relationship between AI cooperation intensity and absorptive capacity, but it also makes visible the considerable heterogeneity in initial conditions across ASEAN members. This descriptive picture motivates the fixed-effects specifications that follow; the regressions aim to isolate the cooperation-capability association net of time-invariant country differences, shared year shocks and observable controls.

**Figure 2.**
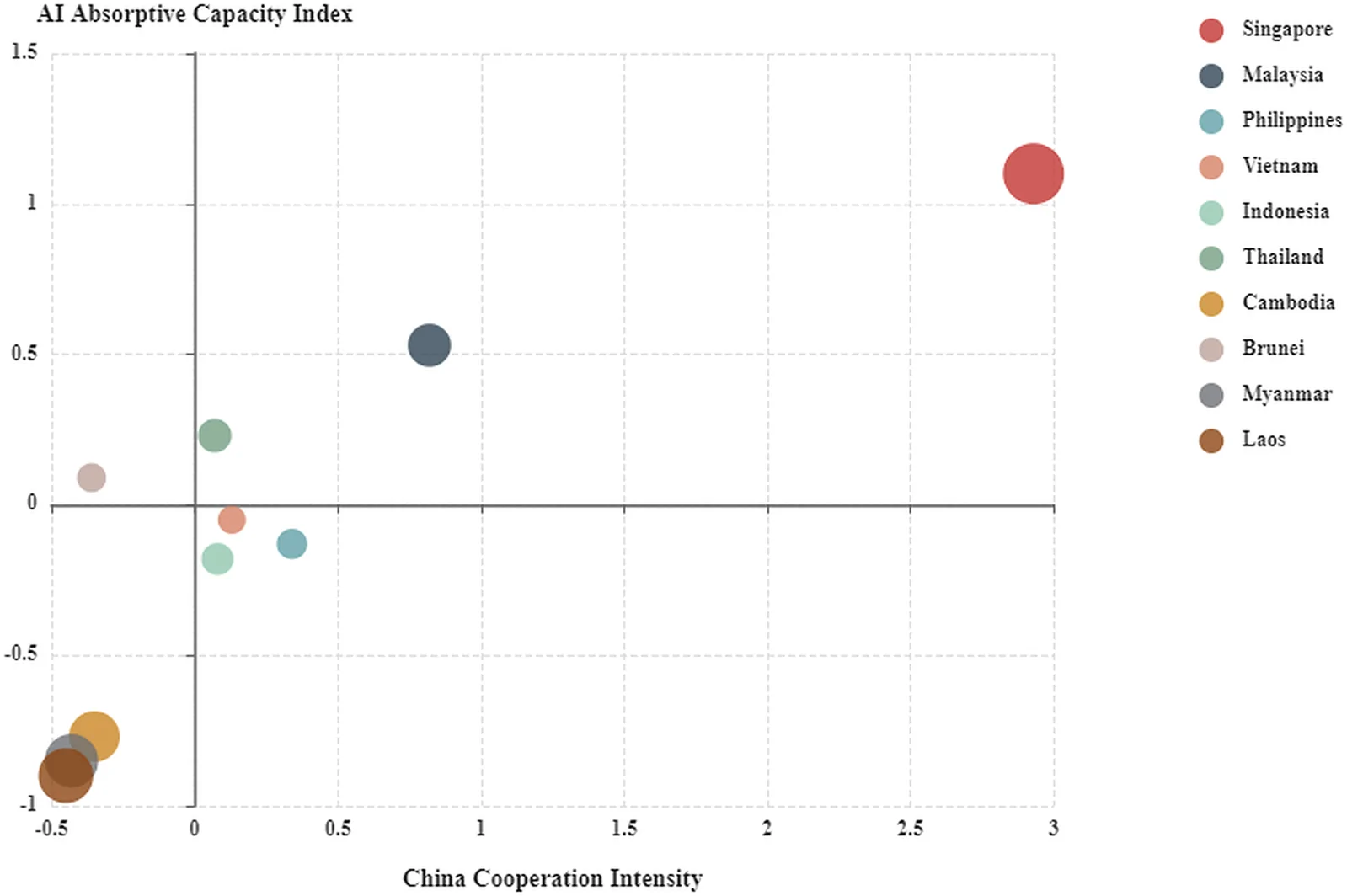
China-related AI cooperation intensity and AI absorptive capacity in ASEAN economies.

### 4.2. Baseline estimates

Table 3 reports the baseline fixed-effects estimates. Model 1 includes only the composite AI cooperation variable and the full set of controls and fixed effects. Model 2 adds the China-partner indicator, the low-initial-industrial-foundation dummy and the relevant two-way and three-way interactions. The central finding is the positive coefficient on bilateral AI cooperation intensity, which is statistically significant at the 1% level in both specifications. In model 1, the estimated coefficient is 0.0471 (*p* < 0.01). In model 2, which incorporates the heterogeneity terms, the coefficient rises slightly to 0.0524 and remains significant at the 1% level. The stability of this estimate across specifications indicates that stronger bilateral AI cooperation is consistently associated with higher absorptive capacity in ASEAN economies. This finding supports H1.

**Table 3.** Baseline regression results.

variables	model 1	model 2
BAIC	0.0471^***^	0.0524^***^
	(0.0137)	(0.0150)
LNGDPPC	0.242	0.237
	(0.178)	(0.181)
OPEN	–0.0118	–0.0174
	(0.129)	(0.135)
GOVEFF	−0.0621	–0.0619
	(0.0935)	(0.0937)
FDI	–4.66e-12^***^	–4.63e-12^***^
	(1.08e-12)	(1.08e-12)
CHN × BAIC		–0.0279^***^
		(0.00855)
LOWIND		–0.443^*^
		(0.239)
LOWIND × BAIC		0.0153
		(0.0742)
CHN × LOWIND × BAIC		0.0639^*^
		(0.0371)
ASEAN FE	yes	yes
partner FE	yes	yes
year FE	yes	yes
observations	1397	1397
*R*^2^	0.959	0.959

Notes: Cluster-robust standard errors in parentheses, clustered at the country-year level. *p < 0.10, ***p < 0.01.

Substantively, the results are consistent with the view that cross-border AI linkages can serve as channels through which external knowledge, technology and resources reinforce domestic conditions for innovation, application and industrial support. However, the fixed-effects design controls for time-invariant unobservable factors and shared time trends, not for time-varying confounders. The estimates, therefore, capture a conditional association rather than a well-identified causal effect. Among the controls, only foreign direct investment is negative and statistically significant across both specifications. The other control variables do not display stable patterns. Since the paper is primarily concerned with the cooperation terms, the discussion that follows concentrates on the average association captured by bilateral AI cooperation intensity and on the heterogeneity of this association across country groups.

### 4.3. Heterogeneous associations

Model 2 in table 3 provides a more differentiated picture. The coefficient on the interaction between the China-partner dummy and bilateral AI cooperation intensity is −0.0279, negative and statistically significant. On its own, this estimate indicates that China-related cooperation does not carry a general premium across the full ASEAN sample. If anything, the average China-related association appears weaker than that of other bilateral partners.

The pattern shifts once initial industrial conditions are taken into account. The triple interaction among China, low initial industrial foundation and bilateral AI cooperation intensity is positive, with a coefficient of 0.0639 that reaches significance at the 10% level. Interpreting these coefficients jointly: for ASEAN economies with stronger initial industrial foundations, the China-related association is roughly the main cooperation effect (0.0524) plus the China interaction (−0.0279), yielding a net coefficient of approximately 0.0245. For economies with weaker initial foundations, the triple interaction adds another 0.0639, bringing the net China-related association to approximately 0.0884. The difference between these two net coefficients is driven by the triple interaction term.

This pattern is consistent with a conditional, compensatory role for China-related cooperation rather than a uniform advantage. In economies where domestic industrial support conditions were initially thinner, the same level of China-related cooperation intensity appears associated with a larger increment in absorptive capacity. From an absorptive-capacity perspective, this makes substantive sense: external linkages can carry technical interfaces, application pathways and supporting resources that are harder to access through purely domestic channels in economies with weaker industrial ecosystems. The marginal contribution of external cooperation is plausibly larger precisely where domestic alternatives are scarcest. That said, two caveats warrant emphasis. First, the triple interaction is significant only at the 10% level, so the statistical confidence in this heterogeneity pattern is modest. Second, as the robustness analysis in §4.5 will show, the triple interaction loses significance when the sample is restricted to the observed-data period. The China-related heterogeneous finding should therefore be treated as suggestive rather than definitive, a point we return to below.

### 4.4. Results by absorptive-capacity dimension

The baseline index aggregates three conceptually distinct dimensions of absorptive capacity: innovation foundations, application foundations, and industrial support conditions. To examine whether the baseline association is concentrated in particular dimensions, table 4 re-estimates the model using each dimension as a separate dependent variable. The results indicate a positive and statistically significant association across all three components. The coefficient on bilateral AI cooperation intensity is 0.0496 for innovation foundations (*p* < 0.01), 0.0434 for application foundations (*p* < 0.01) and 0.0481 for industrial support conditions (*p* < 0.05). The overall picture is one of broad-based association: bilateral AI cooperation intensity is linked not only to the composite index but to each of its constituent layers. At the same time, the precision of the estimates differs across dimensions. The coefficients for innovation and application foundations are more tightly estimated, while the industrial support dimension shows somewhat larger standard errors. This pattern aligns with substantive expectations. Innovation-related indicators (R&D expenditure and tertiary enrolment) and application-related indicators (Internet access and broadband penetration) are more directly connected to the knowledge-exchange and collaborative activities that bilateral AI cooperation entails. Industrial support conditions (ICT service exports and logistics performance) reflect cumulative infrastructure and institutional development, which may respond more slowly to external cooperation inputs. The component results reinforce the baseline finding and rule out the concern that the composite-index result is driven by a single dimension. Bilateral AI cooperation intensity is associated with capability formation across the full architecture of the domestic innovation system.

**Table 4.** Results by absorptive-capacity dimension.

variables	model 1 INNOV	model 2 APPL	model 3 INDUS
BAIC	0.0496^***^	0.0434^***^	0.0481^**^
	(0.0154)	(0.0137)	(0.0215)
controls	yes	yes	yes
ASEAN FE	yes	yes	yes
partner FE	yes	yes	yes
year FE	yes	yes	yes
observations	1397	1397	1397
*R*^2^	0.952	0.959	0.881

Notes: Cluster-robust standard errors in parentheses. **p < 0.05, ***p < 0.01.

### 4.5. Robustness checks

Table 5 reports two sets of sensitivity checks. The first restricts the sample to the 2015–2023 period, removing years for which a subset of control variables required limited interpolation. The second applies 1% winsorization to the dependent variable, the core explanatory variable and the continuous controls. The restricted-sample results preserve the core finding. In the baseline specification, the coefficient on bilateral AI cooperation intensity is 0.0241 (*p* < 0.05). In the specification with heterogeneity terms, it is 0.0218 (*p* < 0.05). The average positive association between AI cooperation and absorptive capacity is therefore not an artefact of including the interpolated years. The restricted sample also clarifies the limits of the heterogeneous result. The interaction between the China-partner dummy and bilateral AI cooperation remains negative and statistically significant, but the triple interaction becomes small and loses statistical significance. This is consistent with the earlier caution: the China-related heterogeneity pattern, while suggestive in the full sample, is less stable under stricter sample constraints. The main bilateral result, by contrast, survives the restriction intact. The winsorized estimates tell the same story. In the winsorized baseline specification, the coefficient on bilateral AI cooperation intensity is 0.0509 (*p* < 0.01). In the winsorized specification with heterogeneity terms, it is 0.0552 (*p* < 0.01). The China interaction remains negative and significant, and the triple interaction is positive and significant at the 10% level. Across both robustness exercises, the most stable and reliable finding is the positive relationship between bilateral AI cooperation intensity and AI absorptive capacity. This relationship survives sample restriction, outlier treatment and the inclusion of interaction terms. By contrast, the China-related heterogeneous pattern is sensitive to specification and sample window. The weight of the evidence supports a strong average bilateral association and a narrower, more tentative conditional pattern for China-related cooperation.

**Table 5.** Robustness checks.

variables	model 1 2015–2023	model 2 2015–2023	model 3 winsorized	model 4 winsorized
BAIC	0.0241^**^	0.0218^**^	0.0509^***^	0.0552^***^
	(0.0113)	(0.0106)	(0.0143)	(0.0154)
CHN × BAIC		–0.00903^***^		–0.0261^***^
		(0.00309)		(0.00889)
LOWIND		–0.303		–0.445^*^
		(0.277)		(0.239)
CHN × LOWIND × BAIC		0.0135		0.0622^*^
		(0.0258)		(0.0371)
controls	yes	yes	yes	yes
ASEAN FE	yes	yes	yes	yes
partner FE	yes	yes	yes	yes
year FE	yes	yes	yes	yes
observations	1143	1143	1397	1397
*R*^2^	0.976	0.976	0.959	0.960

Notes: Cluster-robust standard errors in parentheses. Models 1 and 2 restrict the sample to 2015–2023. Models 3 and 4 apply 1% winsorization. *p < 0.10, **p < 0.05, ***p < 0.01.

## 5. Conclusion and policy implications

### 5.1. Conclusion

Whether cross-border technology cooperation strengthens domestic capability in recipient economies, is a question with growing policy urgency. The World Intellectual Property Organization's 2026 report identifies absorptive capacity as one of four critical factors determining how quickly and widely technologies diffuse across countries, alongside technology traits, information flows and supportive policy frameworks [30]. This paper took up the empirical side of that question by examining whether bilateral AI cooperation is associated with stronger AI absorptive capacity in ASEAN economies, and whether this association varies by partner and by initial domestic conditions. Drawing on absorptive-capacity theory [1,12], we constructed a composite index covering innovation foundations, application foundations and industrial support conditions for 10 ASEAN economies, and linked it to bilateral AI cooperation intensity measured through co-publications, co-patents and investment linkages. Using a bilateral panel with fixed-effects specifications, we obtained a clear central finding and a more qualified secondary finding.

The central finding is a positive association between bilateral AI cooperation intensity and AI absorptive capacity. The association is significant at the 1% level, stable across specifications, and survives both sample restrictions and winsorization. It is present across all three constituent dimensions of the index, with innovation and application foundations more tightly estimated than industrial support conditions. This broad-based pattern indicates that cross-border AI linkages are associated with capability formation across knowledge generation, technology application and industrial embedding, rather than with isolated pockets of improvement. The finding extends prior work on absorptive capacity, which has focused primarily on FDI and trade as channels of international technology diffusion [9,19], to the domain of policy-mediated bilateral cooperation. The cooperation channel combines knowledge exchange, collaborative production and institutional partnership in ways that market-mediated flows alone do not capture.

The secondary finding concerns heterogeneity. Our estimates do not support the claim that China-related cooperation is uniformly more favourable than cooperation with other partners. The two-way China interaction is negative and significant, indicating no general China premium across the full ASEAN sample. A more favourable China-related association appears specifically in economies with weaker initial industrial foundations, where the triple interaction is positive but only at the 10% significance level, and it loses significance under sample restrictions. This conditional and fragile pattern parallels recent evidence that innovation and technology effects are not uniform across country income groups. Oluwaseyi Musibau *et al.* [31], studying Oceanic-Asian economies, find that innovation reduces emissions in some country groups while increasing them in others through rebound effects, and that its moderating role is insignificant in low-income settings where technical and managerial skills are thinner. The parallel with our findings is instructive: external technological inputs, whether innovation or cooperation, do not produce uniform effects across heterogeneous economies.

The China-related result runs counter to expectations sometimes implicit in policy discourse that Chinese technology cooperation carries distinctive developmental advantages. Deng *et al.* [32] show that negative China-related public opinion is associated with decreased Chinese exports through consumer preference, policy uncertainty and political conflict channels. Their finding that China's international economic effects are mediated by partner-country conditions aligns with our conclusion that China-related cooperation effects are not inherent to the partnership itself but contingent on domestic absorptive conditions. The value of external linkages depends less on the partner's identity than on how the cooperation interacts with the receiving economy's capabilities.

The paper's contribution is to demonstrate that the absorptive-capacity framework travels productively from its original FDI and firm-level settings to the domain of bilateral technology cooperation at the country level. By shifting the analytical lens from market-mediated flows to policy-mediated cooperation, and from firm-level to country-level outcomes, the paper opens inquiry at the intersection of the absorptive-capacity literature, the regional cooperation literature and the emerging work on AI and development. The results suggest that the developmental significance of AI cooperation depends less on the volume of cooperation than on whether external knowledge is embedded into domestic systems of learning, application and industrial adaptation, a conclusion consistent with the World Intellectual Property Organization emphasis on absorptive capacity as a precondition for technology diffusion to generate inclusive development outcomes [30].

### 5.2. Policy implications

The findings carry implications for the design and evaluation of AI cooperation policy in the Asia-Pacific, though we distinguish between implications that follow directly from the empirical results and those that follow as reasoned inference.

The most robust implication follows from the central finding. The positive and stable association between bilateral AI cooperation and absorptive capacity suggests that the developmental value of cooperation should not be assessed by the volume of agreements, the scale of investment or the diplomatic visibility of partnerships. These metrics capture inputs. A more informative criterion is whether cooperation contributes to the domestic conditions, including research capacity, human capital, digital infrastructure and industrial support, that allow external knowledge to be absorbed and used. For ASEAN policymakers, this means that AI cooperation initiatives are more likely to produce durable gains when they are embedded in broader domestic strategies for education, research and digital development. Cooperation that increases the flow of external knowledge without strengthening the domestic conditions for absorbing it risks producing short-term visibility without long-term capability.

The dimension-level results offer practical guidance on sequencing. Innovation and application foundations show the most precisely estimated associations with cooperation intensity. In operational terms, the earliest measurable policy returns are likely to appear in research collaboration, human capital formation and digital readiness. Broader industrial upgrading, captured by the industrial support dimension, may take longer to materialize, consistent with the gradual diffusion patterns Brynjolfsson *et al.* [16] document for general-purpose technologies. Evaluation frameworks for AI cooperation should therefore differentiate among layers of capability development and avoid the expectation of uniform and simultaneous progress across all dimensions. Measuring the success of cooperation policy by industrial outcomes alone, without tracking the intermediate indicators of research capacity and digital readiness, would risk prematurely dismissing policies that are in fact building the foundations for longer-term gains.

The heterogeneous results counsel against uniform cooperation templates. ASEAN economies vary enormously in their digital readiness, innovation resources and industrial conditions. The data suggest that economies with weaker initial industrial foundations may derive larger marginal capability gains from the same level of cooperation intensity. For these economies, cooperation design may usefully emphasize applied training, infrastructure support, technical interfaces and local institutional embedding alongside knowledge exchange and research collaboration. For economies with stronger existing foundations, cooperation may be more productively oriented towards advanced research collaboration, standards development and specialized applications. The broader point is not that one partner or one type of cooperation is superior, but that cooperation design should be differentiated. A uniform template is unlikely to be equally effective across an economically heterogeneous region.

The China-related findings warrant measured interpretation. The data do not support the claim that Chinese cooperation is uniformly more beneficial than cooperation with other partners. Across the full ASEAN sample, the average China-related association is not stronger than the average bilateral association. A more favourable China-related pattern appears specifically in economies with weaker initial industrial foundations, and this pattern is statistically fragile. The policy implication is not that Chinese cooperation lacks value; the data show that it is positively associated with absorptive capacity in the relevant subsample. Rather, claims about its distinctive developmental role should be careful and context-specific. Chinese cooperation appears to function as a complement to thin domestic industrial ecosystems rather than as a universally superior channel.

One broader implication concerns evaluation criteria. Regional digital cooperation is typically evaluated by the scale of projects, the amount of investment or the level of diplomatic attention attracted. Our results indicate that these criteria capture inputs, not outcomes. A more informative evaluation would ask whether cooperation strengthens innovation foundations, broadens application readiness and supports industrial embedding, and would track these dimensions over time, recognizing that progress in each dimension follows different timelines. This reorientation from input-based to capability-based evaluation is especially relevant in the ASEAN context, where cooperation initiatives proliferate but systematic evidence on their developmental returns remains scarce.

### 5.3. Limitations

Four limitations warrant attention. First, the dependent variable is a composite index that smooths over sector-specific and subnational variation; future work with disaggregated capability measures would address this directly. Second, the fixed-effects design does not address time-varying confounders, such as concurrent improvements in digital endowments driving both cooperation and absorptive capacity. Instrumental-variable or dynamic panel strategies would tighten causal identification. Third, the China-related heterogeneity finding depends on sample composition and loses significance under stricter sample constraints, underscoring its provisional nature. A longer time series and data on the substantive content of cooperation would be needed for greater confidence. Fourth, the cooperation measure aggregates co-publication, co-patenting and investment linkages, masking potentially heterogeneous effects across cooperation types. Disaggregating by channel is a natural next step.

## Supplementary Material



## Data Availability

The data, code and supporting material required to reproduce the results of this study are publicly available at Zenodo [33]. The repository contains the full estimation dataset covering 10 ASEAN economies over 2015–2025. The same material is also provided as supplementary material [34].
